# Ameliorating effects of ramelteon on oxidative stress, inflammation, apoptosis, and autophagy markers in methotrexate-induced cerebral toxicity

**DOI:** 10.22038/IJBMS.2022.62955.13913

**Published:** 2022-10

**Authors:** Rahime Aslankoc, Mehtap Savran, Duygu Kumbul Doğuç, Murat Sevimli, Hale Tekin, Mine Kaynak

**Affiliations:** 1 Süleyman Demirel University, Faculty of Medicine, Department of Physiology, Isparta, Turkey; 2 Süleyman Demirel University, Faculty of Medicine, Department of Pharmacology, Isparta, Turkey; 3 Süleyman Demirel University, Faculty of Medicine, Department of Biochemistry, Isparta, Turkey; 4 Süleyman Demirel University, Faculty of Medicine, Department of Histology and Embryology, Isparta, Turkey; 5 Süleyman Demirel University, Graduate School of Natural and Applied Sciences, Department of Bioengineering, Isparta, Turkey

**Keywords:** Apoptosis, Autophagy, Cerebral toxicity, Inflammation, Methotrexate, Oxidative stress, Ramelteon

## Abstract

**Objective(s):**

Methotrexate (MTX) is a widely used chemotherapeutic agent that, however, is known to have serious side effects such as neurotoxicity. In the present study, we aimed to evaluate the possible favorable effects of ramelteon (RMLT) on MTX-induced cerebral toxicity.

**Materials and Methods:**

Thirty-two male Wistar albino rats were divided into four groups: Control group, MTX group (20 mg/kg MTX, IP, single dose), MTX+RMLT group (20 mg/kg MTX, IP, single dose + 10 mg/kg RMLT, by gavage, 7 days), and RMLT group (10 mg/kg RMLT, by gavage, 7 days).

**Results:**

In the MTX group, increased levels of total oxidant status (TOS) and oxidative stress index (OSI) levels and decreased levels of total antioxidant status (TAS) level were observed. RMLT significantly reversed oxidative stress parameters. Real-time PCR analysis revealed that MTX increased the expressions of Beclin-1 and autophagy-related gene 12 (ATG12). These expressions were significantly decreased by RMLT. Vacuolar changes, apoptotic cells, and inflammatory cell infiltration induced by MTX were ameliorated by RMLT treatment. Increased tumor necrosis factor-α (TNF- α) and Caspase-3 activities induced by MTX were returned to their normal levels by RMLT.

**Conclusion:**

All our results demonstrate that RMLT alleviates the harmful effects of MTX on the cerebral cortex tissue. Therefore, RMLT may be considered for supportive therapy for preventing side effects of MTX in patients needing MTX therapy.

## Introduction

Methotrexate (MTX) is an antimetabolite agent that binds to and inhibits the enzyme dihydrofolate reductase (DHFR), preventing the conversion of dihydrofolate to tetrahydrofolate and blocking DNA synthesis (1). MTX is a chemotherapeutic agent that is widely used in the treatment of some types of cancer (such as acute lymphoblastic leukemia and lymphoma) as well as inflammatory disorders, such as rheumatoid arthritis (2). This drug can cause various side effects, such as cardiological (e.g., arrhythmia), gastrointestinal (e.g., nausea and vomiting), and psychological disorders (e.g., depression) as well as immunosuppression (3). 

Under normal physiological conditions, there is a balance between the production of reactive oxygen species (ROS) and antioxidant enzyme activity in cells. In the event of a decrease in the number of antioxidant enzymes, the balance is disturbed, and ROS causes damage to the cell (4). MTX increases the formation of ROS. This leads to cell cycle arrest and cell death in the central nervous system (5). In recent years, treatment with MTX has been reported to cause severe neurotoxicity in the hippocampus, reduction of hippocampal neurogenesis, and alterations in memory ability (6, 7). Autophagy has been considered a cell survival mechanism and intracellular control mechanism in response to cellular stressors. Autophagy is induced in conditions that cause cellular stress, such as nutrient and growth factor deficiencies, and is responsible for degradation of damaged organelles, long-lived proteins, and protein aggregates (8). In case of nutrient starvation, unneeded proteins are degraded and the amino acids are recycled for the synthesis of proteins that are essential for survival. Furthermore, autophagy can be triggered by hypoxia, nutrient depletion, or cellular stress induced by ROS (9). Extracellular signals that are sensed by specific transmembrane receptors cause apoptotic cell death. While extracellular stress signals trigger apoptosis by activating the TNF-α pathway, intracellular stress signals, such as DNA damage, oxidative stress, or cytosolic calcium increase, activating Caspase-3, and causing apoptosis (10). Autophagic cell death can be suppressed by inhibition of the autophagic pathway by some chemicals or genetic tools (such as RNAi or gene mutations targeting key autophagic modulators, such as autophagy-related gene 12 (ATG12) and Beclin 1 (11). 

Ramelteon (RMLT) is a selective melatonin receptor (MT1 and MT2) agonist used to treat insomnia (12). RMLT has advantages over hypnotic drugs in having no affinity for benzodiazepine, dopamine, opiate, or serotonin receptor binding sites; having no side effects on cognitive and psychomotor functions, and being non- addictive (13). RMLT is a more potent melatonin agonist as it is more effectively due to its longer duration of action. Moreover, it can prevent neurotoxicity as well as improve sleep quality in patients with neurodegenerative diseases (14). 

In recent years, autophagy has been shown to be one of the primary mechanisms of cell death in addition to apoptosis. Therefore, we hypothesized that RMLT may show protective effects in MTX-induced cerebral cortex damage. The current study was designed to evaluate the protective effects of RMLT against MTX-induced cerebral neurotoxicity. Thus, several assessments of toxicity TOS and TAS levels, TNF-α for inflammation, Caspase-3 for apoptosis, Beclin-1 and ATG12 mRNA expressions by qRT-PCR for autophagy were performed following MTX exposure**.**

## Materials and Methods

***Animals***

The experimental design was approved by the Animal Experiments Local Ethics Committee of Süleyman Demirel University, Turkey (Ethic No: 06/13, 11.09.2020 ). The study was conducted in accordance with the animal research guidelines of the National Institute of Health. 

***Study groups***

The study was performed on a total of thirty-two male Wistar albino rats weighing 200–300 grams. All rats were housed in standard housing facilities (temperature 22–23 °C, 60% ± 5% humidity, and 12 hr light/dark cycle). The animals were provided with *ad libitum* access to standard laboratory chow and tap water. The rats were randomly divided into four groups (n=8, each) as follows; 

1. The Control group received 0.1 ml saline by oral gavage for 7 days. In addition, a single dose of saline was administered intraperitoneally (IP) on day 2.

2. MTX group received a single dose of MTX (20 mg/kg) on the second day, IP ( MTX 50 mg/ml flk, Kocak, Turkey), and 0.1 ml saline was given for 7 days by oral gavage (15). 

 3. MTX + RMLT group received a single dose of MTX (20 mg/kg) on the second day, IP , and 0.1 ml RMLT (10 mg/kg) was given for 7 days by oral gavage (16). 

4. RMLT group received a single dose of saline (0.1 ml) on the second day, IP, and 0.1 ml RMLT (10 mg/kg) was given for 7 days by oral gavage.

Twenty-four hours after the last drug administration, all rats were sacrificed via intraperitoneal injection of a ketamine (90 mg/kg, Alfamine, Alfasan IBV) and xylazine (10mg/kg, Alfazin, Alfasan IBV) combination. Following anesthesia, the cerebral cortex was removed and half was stored at -20 °C for analysis of the total antioxidant status, total oxidant status, oxidative stress index, and Beclin-1 and ATG12 gene expressions. The other half of the tissue was fixed in 10% neutral buffered formalin for histopathological and immunohistochemical analyses to evaluate the expression of Caspase-3 and TNF-α proteins.

***Biochemical analyses***

*Measurement of oxidative stress parameters in the cerebral cortex*

Cerebral cortex tissue samples were homogenized using an Ultra Turrax Janke & Kunkel T-25 homogenizer (IKA®-Werke, Germany) for the analysis of oxidant-antioxidant parameters. Total antioxidant status (TAS) and total oxidant status (TOS) were measured spectrophotometrically (Beckman Coulter AU 5800, Beckman Coulter, USA) using the commercial test kits (Rel Assay Diagnostics, Gaziantep, Turkey). OSI was calculated using the formula OSI = TOS/TAS (17). 

The following method was used for TAS analysis, Fe2+-o-dianisidine complex creates a Fenton-type reaction with hydrogen peroxide to form OH radicals. These potent reactive oxygen species react with the colorless o-dianisidine molecule at low pH to form yellow-brown dianisidyl radicals. Dianisidyl radicals participate in advanced oxidation reactions and increase color formation. However, antioxidants in samples suppress oxidation reactions and stop color formation. In this study, this reaction was measured spectrophotometrically by an automated analyzer. The results were expressed as millimolar Trolox equivalent per liter (17). 

The following method was used for TOS analysis**:** oxidants present in the sample oxidize the ferrous ion-o’dianisidine complex to ferric ion. The glycerol in the environment accelerates this reaction and triples it. Ferric ions form a colored complex with xylenol orange in an acidic medium. The color intensity correlated with the number of oxidants in the sample was measured spectrophotometrically. 

The assay was calibrated with hydrogen peroxide, and the results were expressed as hydrogen peroxide equivalents per liter (µmol H_2_O_2_ Eqv/l) (18). 

***Reverse transcription-polymerase chain reaction (RT-qPCR)***

*Total RNA extraction and purification*

Total RNA was extracted from rat tissues using a TRIzol^TM^ Reagent Monarch Total RNA isolation kit (New England BioLabs) following the manufacturer’s instructions. RNA concentration and purity were detected by a MySPEC microvolume spectrophotometer (VWR)

*cDNA synthesis*

1 μg RNA was reverse-transcribed by an iScript cDNA Synthesis kit using oligo dT primers (Bio-Rad Laboratories, Hercules, CA). The reaction mixture was incubated at 25 °C for 5 min, afterward, at 46 °C for 20 min, and finally, at 95 °C for 1 min.

*qRT-PCR*

Real-time PCR amplification was performed using iTaq Universal SYBR Green Supermix (Bio-Rad Laboratories, Hercules, CA, USA) according to the manufacturer’s instructions, and fluorescence was detected with a CFX96 instrument (Bio-Rad Laboratories, Hercules, CA, USA). Specific primers were designed to amplify Beclin-1 (Forward 5’-CTCAGGAGAGGAGCCATTTATT-3’’, Reverse 5’-CCC GATCAGAGTGAAGCTATT-3’’) and ATG12 (Forward 5’-CCACACTTCTGTTTCCTGTTTC-3’’, Reverse 5’- 5’-GG AATCAACACAGTGCCAATC-3’’). PCR was performed in triplicates for each cDNA sample. GAPDH was used for normalization. The PCR reaction conditions were as follows: initial denaturation at 95 °C for 10 min, followed by 40 cycles of 10 sec at 95 °C and 30 sec at 60 °C. The total reaction volume was 25 µl, and 100ng cDNA was used as a template. Relative quantification of gene expression was carried out using the comparative ΔΔCt method. PCR products were identified by a melting curve analysis to find the specificity of amplification. All results are presented in the graph as a fold change.

***Histological analyses***

*Histopathological analysis*

The cerebral cortex taken from animals was fixed in a 10% buffered formaldehyde solution (Tekkim). Before tissue processing, the samples were washed with running tap water overnight, then, dehydrated in graded ethanol, cleared with xylene, and embedded in paraffin. Sections of 4–5 μm thickness were cut from paraffin blocks (RM2125RTS, Leica, Germany) and stained with hematoxylin and eosin (H&E)(Sigma). A light microscopic examination (Eclipse Ni-U; Nikon) was performed to identify the presence of vacuolar changes, necrotic cells, and inflammatory cell infiltration (19). The findings were graded according to a modified semi-quantitative analysis, and assigned a score ranging from (0): no findings, (1): low level, (2): moderate level, (3): severe level (20). 

*Immunohistochemical analysis*

The activity of Caspase-3 and TNF-α was detected immunohistochemically. For this purpose, the sections were first deparaffinized and dehydrated. Afterward, they were incubated with 3% hydrogen peroxide, Ultra-V Block (ThermoFisher), primary antibodies (Bioss), secondary antibodies (ThermoFisher), and streptavidin peroxidase (ThermoFisher). Positive cells were stained with DAB, and counterstaining was done with hematoxylin. The sections were examined using a light microscope (Eclipse Ni-U; Nikon), and immunoreactivity levels were scored using a modified semi-quantitative scale as follows: (0), no staining; (1), weak staining; (2), moderate staining; and (3), intense staining (20). 

***Statistical analysis***

GraphPad Prism 9 software was used for histological analysis, and SPSS package, version 22.0 (SPSS Inc., Chicago, IL, USA) was used for other biochemical analyses. One-way analysis of variance (ANOVA) was used to compare the groups, and Tukey multiple comparison tests were performed. Variables were presented as mean ± standard deviations (*SD*). *P*-value<0.05 was considered statistically significant. 

## Results

***Biochemical results***

*Oxidative stress parameters in the cerebral cortex*

**Figure 1:**
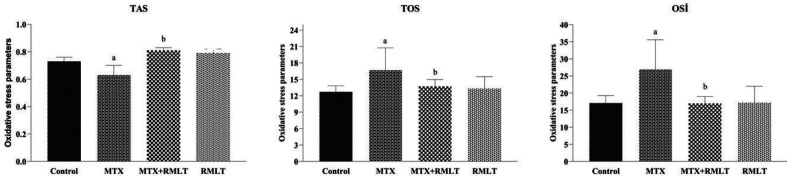
Oxidative stress markers of the cerebral cortex. Data were expressed as means± SD. The oxidative stress markers were compared between groups by one-way ANOVA (with* post hoc *LSD test). a: compared with the control group; *P*=0.003 for TOS, *P*=0.001 for OSI, *P*=0.015 for TAS. b: compared with the MTX group; *P*=0.022 for TOS, *P*=0.001 for OSI, *P*=0.001 for TAS

TAS: Total anti-oxidative status; TOS: Total oxidative status, OS I: Oxidative stress index

In the MTX-administered group, mean TOS level and OSI index were increased (*P*=0.003 and *P*=0.001, respectively), whereas mean TAS level was significantly decreased (*P*=0.015) compared with the control group. On the other hand, in the RMLT-treated group, mean TOS level and OSI index were decreased (*P*=0.022 and *P*=0.001, respectively), and mean TAS level was significantly increased (*P*=0.001) compared with the MTX group. Only in the RMLT-administered group, no significant difference was found between mean TOS, TAS levels, and OSI index compared with the control group (Figure1).

*Beclin-1 and ATG12 mRNA levels in the cerebral cortex*

**Figure 2:**
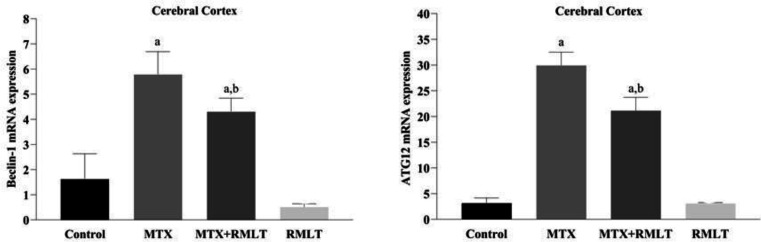
mRNA expression of Beclin-1 and ATG12. Data were expressed as means± SD. The expression data were compared between groups by one-way ANOVA (with *post hoc* LSD test). a: compared with the control group; *P*=0.001 and 0.007, b: compared with the MTX group; *P*=0.034 Beclin-1. a: compared with the control group; *P*=0.001 and P=0.001 b: compared with the MTX group; *P*=0.035 for ATG12

ATG12: Autophagy-related gene; MTX; Methotrexate; RMLT: Ramelteon

The mRNA expression of Beclin-1 was significantly increased in MTX and MTX+RMLT groups compared with the control group (*P*=0.001 and *P*=0.007, respectively). When MTX+RMLT and RMLT groups were compared with the MTX group separately, a significant decrease in the mRNA expression of Beclin-1 was found in both groups (*P*=0.034 and *P*=0.001, respectively) (Figure 2). 

The mRNA expression of ATG12 was significantly increased in MTX and MTX+RMLT groups compared with the control group (*P*=0.001 and *P*=0.001, respectively). When MTX+RMLT and RMLT groups were compared with the MTX group separately, a significant decrease in the mRNA expression of ATG12 was found in both groups (*P*=0.035 and *P*=0.001, respectively) (Figure 2). 

***Histological results***

*Histopathology*

**Figure 3:**
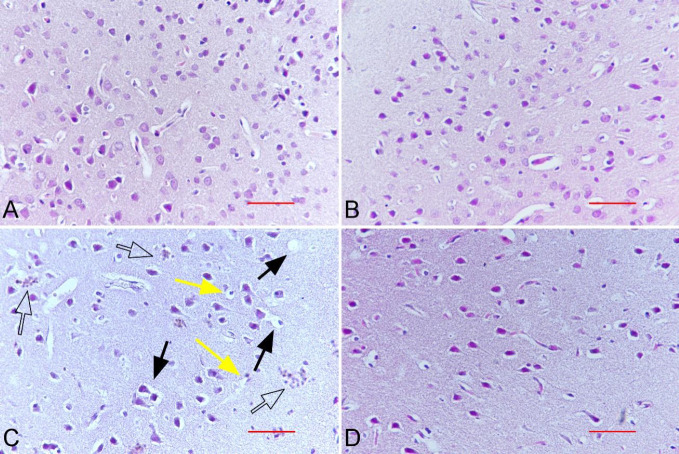
Rat cerebral cortex sections A, B) normal brain histology in control and RMLT groups. C) vacuolar changes (black arrow), apoptotic cells ( yellow arrow), inflammatory cell infiltrations (arrow outline) in MTX groups. D) improvement in histopathological findings and normal histological appearance in the MTX+RMLT group. H & E staining, x40, scale bar=50 µm)

The histopathological findings are presented in Table 1. Normal cerebral cortex histology was observed in the control group samples (Figure 3A). There was no difference in findings between RMLT and the control group where normal cerebral cortex histology was observed (*P*>0.05 for all findings) (Figure 3B). Significant histopathological findings including vacuolar changes, apoptotic cells, and inflammatory cell infiltration were observed in the MTX group compared with the control group (*P*=0.001 for all findings) (Figure 3C). A significant improvement in pathological findings was observed in the MTX+RMLT group compared with the MTX group (*P*=0.001 for all findings) (Figure 3D).

*Immunohistochemistry*

**Figure 4:**
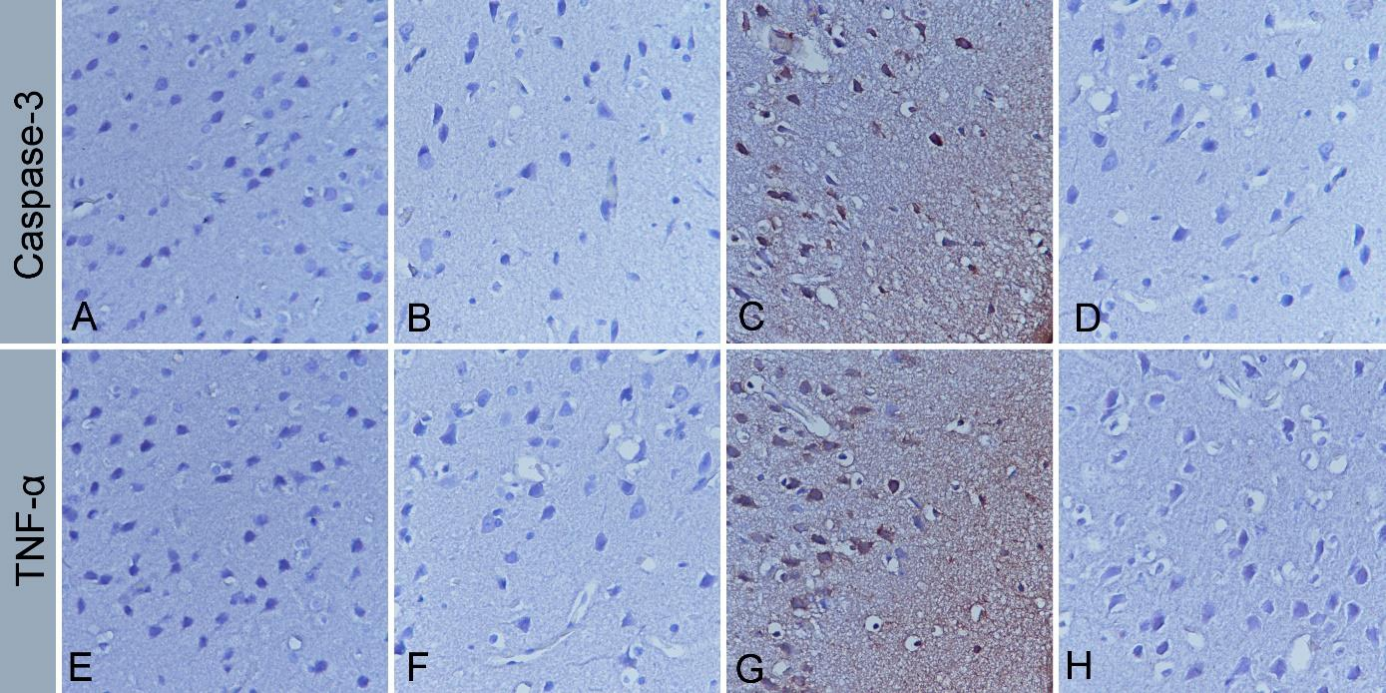
Immunohistochemical stainings. Caspase-3; no immunoreactivity was observed in control (A), RMLT (B) and MTX+RMLT (D) groups; moderate staining in MTX group (C). TNF-α; no immunoreactivity was observed in control (E), RMLT (F), and MTX+RMLT (H) groups; moderate staining in MTX group (G). Streptavidin biotin peroxidase method

The results of immunohistochemistry are presented in Table 2. Caspase-3 immunoreactivity in the cerebral cortex tissue was not observed in the control group (Figure 4A). Caspase-3 immunoreactivity was negative in RMLT (Figure 4B) and MTX+RMLT groups (Figure 4D). However, compared with the control group, moderate staining was observed in the MTX group (*P*=0.001) (Figure 4C). The results of TNF-α immunoreactivity results were similar to those of Caspase-3. TNF-α immunoreactivity was not observed in the control (Figure 4E), RMLT (Figure 4F), and MTX+RMLT (Figure 4H) groups, while moderate immunoreactivity was observed in the MTX group (*P*=0.001) (Figure 4G).

**Table 1:** Mean histopathological findings of all groups of rats

**Groups**	**Control** **(mean score)**	**MTX** **(mean score)**	**MTX+RMT** **(mean score)**	**RMT** **(mean score)**
**Histopathological findings**				
Vacuolar changes	**0**	**3**	**1**	**0**
Apoptotic cells	**0**	**3**	**0**	**0**
Inflammatory cell infiltration	**0**	**2**	**0**	**0**

**Table 2:** Mean Immunoreactivity scores of groups for caspase-3 and TNF-α of rats

**Groups**	**Control** **(mean score)**	**MTX** **(mean score)**	**MTX+RMT** **(mean score)**	**RMT** **(mean score)**
**Immunostaining scores**				
Caspase-3	**0**	2	**0**	**0**
TNF-α	**0**	2	**0**	**0**

Immunoreactivity scores of caspase-3 and TNF-α. For both antigens; no immunoreactivity was detected in the control, RMT, and MTX+RMT groups but moderate immunoreactivity was observed in the MTX group (*P*=0.001). (0), no staining; (1), weak staining; (2), moderate staining; and (3), intense staining

## Discussion

Although MTX is widely used as a chemotherapeutic agent, it has adverse side effects on various organs, including the central nervous system. For our study, we chose RMLT treatment to reduce and improve cerebral cortex toxicity caused by MTX and demonstrated that RMLT can reduce cerebral cortex toxicity.

Oxidative stress occurs when the balance between oxidant formation and antioxidant defense is disrupted in favor of oxidants. Increased oxidant production may weaken the antioxidant defense system by causing oxidative damage to cellular lipids, proteins, and DNA. In experimental studies, oxidative stress has been identified as the main mechanism of organ damage (21). MTX increases oxidative stress in many organs, including the brain, and white matter is vulnerable to oxidative stress due to the high content of polyunsaturated fatty acids and a low level of antioxidants (22-24). İncrease in lipid peroxidation induced by MTX causes a deterioration of the the membrane permeability, neurotransmitter-receptor interaction, and induction of cell apoptosis in the cerebral cortex (7, 25). The literature is consistent with the fact that ROS produced by MTX depletes antioxidant activity in the rat cerebral cortex under oxidative stress (19). The data are consistent with our study demonstrating increased TOS levels in the cerebral cortex in MTX-treated rats. In the present research, MTX induced oxidative stress as confirmed by a detected decrease in TAS level in the cerebral cortex. In the literature, the antioxidant property of melatonin is better known than that of RMLT (26). In this context, in a previous study, it was shown that RMLT protects neuronal cells against inflammation and oxidative stress in traumatic brain injury (27)**. **Our results have demonstrated a significant increase in TAS levels in RMLT-treated groups compared with the MTX group**,** along with a significant decrease in TOS levels, supporting the view that RMLT treatment may be effective in protecting against oxidative stress.

Autophagy is an important biological response mechanism regulated by ROS and oxidative stress and is also an intracellular process that delivers the cargo including pathogens to lysosomes for degradation (28). Autophagy can be activated by multiple stress factors, such as hypoxia, nutrient and growth factor deprivation, radiotherapy, and chemotherapy which trigger the accumulation of ROS. Specific ROS**,** often at lower levels, are essential for the efficient activation of stress-induced autophagy. By eliminating intracellular sources of ROS, autophagy is generally thought to be cytoprotective (29). However, prolonged activation of autophagy or specific selective types of autophagy can result in cell death (30). Previous studies have shown that ATG12 and Beclin-1 are important autophagy effectors regulating the initiation and maturation of autophagosomes (31, 32). In a mouse model of Alzheimer’s Disease (AD), reduced Beclin 1 expression has been shown to cause an increase in intraneuronal and extracellular amyloid beta accumulation and accelerated neurodegeneration. In the study, notably, AD-associated amyloid pathology in mice strengthened the idea that it was salvageable by reintroduction of Beclin 1 (33). Therefore, in our study, Beclin-1 and ATG12 mRNA levels were examined in the cerebral cortex tissue of rats treated with MTX. Our results have shown that Beclin-1 and ATG12 mRNA expressions were increased in the MTX-administered group, and these parameters were reversed by RMLT, suggesting that RMLT may regulate autophagy through expressions of Beclin-1 and ATG12.

Based on histopathological findings, such as vacuolar changes, apoptotic cells, and inflammatory cell infiltration in the MTX group, this study has shown that the cerebral cortex damage caused oxidative stress mainly occurred at the histopathological level. RMLT treatment provided significant improvement in histopathological findings compared with the MTX group. Previous studies have demonstrated that MTX causes vacuolar changes and degeneration of neuronal and perineuronal glial cells in the hippocampus and cerebral cortex (34, 35). 

MTX can induce apoptosis through production of ROS, which leads to oxidative stress (36). Caspase-3 activation may play a key role in triggering apoptosis in neuronal cells (37). In a study investigating the neurotoxicity of MTX o hippocampal cells *in vivo* and *in vitro*, it was shown that Caspase-3 expression increased 24 hr after MTX treatment (38). In another study, immunohistochemical analyses have shown that MTX increased Caspase-3 activation in the hippocampus, cerebellum, kidney, and liver tissues (2). In the present study, we have immunohistochemically demonstrated that Caspase-3 expression was increased in the cerebral cortex tissue affected by MTX. RMLT ameliorated the apoptotic changes in the cerebral cortex tissue as shown by decreased Caspase-3 expressions.

In the literature, neuroinflammation has been associated with damage to the central nervous system caused by neurotoxic substances (39). Various inflammatory mediators, such as TNF-α, have been shown to play a central role in neuroinflammation in human and animal research studying chemotherapy exposure (40). In addition, TNF-α plays an important role in the pathogenesis of oxidative stress by inducing ROS production. It stimulates the cellular release of inflammatory mediators and cytokines (41). Therefore, excessive TNF-α release directly activates oxidative stress and the caspase enzyme system, inducing apoptosis and causing brain damage. We found that the elevated level of TNF-α following MTX administration was restored to the normal level by RMLT treatment. Although RMLT likely inhibits the systemic inflammatory response in patients with insomnia (42), it was unclear whether it inhibits the inflammatory response in the MTX model. In this study, we have provided evidence that RMLT treatment significantly ameliorates inflammatory and apoptotic responses induced by MTX by reducing increased levels of both Caspase-3 and pro-inflammatory cytokine TNF-α in the cerebral cortex tissue.

## Conclusion

MTX causes damage to the cerebral cortex by inflammation mediated by TNF-α phosphorylation, in addition to oxidative stress and apoptosis/autophagy. RMLT inhibits these adverse mechanisms and may be a notable drug candidate for MTX-induced organ toxicity. The major future challenge will be to understand the effects of the timing and dosage of ramelteon treatment on inhibiting cell death pathways and underlying mechanisms. The results of this study will prompt new chronotherapeutic strategies for managing side effects of MTX therapy.

## Authors’ Contributions

All authors participated in the design and interpretation of the study, data analysis, and the review of the manuscript. MS and MK Conducted the experiment and collected the tissue, RA was responsible for the data analysis. MS Performed histopathological and immunochemical analyses. DKD and HT Performed biochemical analyzes. RA Wrote the manuscript, and all authors reviewed, read, and approved the article

## Conflicts of Interest

The authors declare that there are no conflicts of interest regarding the publication of this article.
